# Large-scale identification of human genes implicated in epidermal barrier function

**DOI:** 10.1186/gb-2007-8-6-r107

**Published:** 2007-06-11

**Authors:** Eve Toulza, Nicolas R Mattiuzzo, Marie-Florence Galliano, Nathalie Jonca, Carole Dossat, Daniel Jacob, Antoine de Daruvar, Patrick Wincker, Guy Serre, Marina Guerrin

**Affiliations:** 1 UMR 5165 "Epidermis Differentiation and Rheumatoid Autoimmunity", CNRS - Toulouse III University (IFR 30, INSERM - CNRS - Toulouse III University - CHU), allées Jules Guesde, 31073 Toulouse, France; 2 Genoscope and CNRS UMR 8030, rue Gaston Crémieux, 91057 Evry, France; 3 Centre de Bioinformatique Bordeaux, Université V. Segalen Bordeaux 2, rue Léo Saignat, 33076 Bordeaux Cedex, France

## Abstract

**Background:**

During epidermal differentiation, keratinocytes progressing through the suprabasal layers undergo complex and tightly regulated biochemical modifications leading to cornification and desquamation. The last living cells, the granular keratinocytes (GKs), produce almost all of the proteins and lipids required for the protective barrier function before their programmed cell death gives rise to corneocytes. We present here the first analysis of the transcriptome of human GKs, purified from healthy epidermis by an original approach.

**Results:**

Using the ORESTES method, 22,585 expressed sequence tags (ESTs) were produced that matched 3,387 genes. Despite normalization provided by this method (mean 4.6 ORESTES per gene), some highly transcribed genes, including that encoding dermokine, were overrepresented. About 330 expressed genes displayed less than 100 ESTs in UniGene clusters and are most likely to be specific for GKs and potentially involved in barrier function. This hypothesis was tested by comparing the relative expression of 73 genes in the basal and granular layers of epidermis by quantitative RT-PCR. Among these, 33 were identified as new, highly specific markers of GKs, including those encoding a protease, protease inhibitors and proteins involved in lipid metabolism and transport. We identified filaggrin 2 (also called ifapsoriasin), a poorly characterized member of the epidermal differentiation complex, as well as three new lipase genes clustered with paralogous genes on chromosome 10q23.31. A new gene of unknown function, *C1orf81*, is specifically disrupted in the human genome by a frameshift mutation.

**Conclusion:**

These data increase the present knowledge of genes responsible for the formation of the skin barrier and suggest new candidates for genodermatoses of unknown origin.

## Abstract 2

Identification of genes expressed in epidermal granular keratinocytes by ORESTES, including a number that are highly specific for these cells.

## Background

High-throughput genomic projects focusing on the identification of cell- and tissue-specific transcriptomes are expected to uncover fundamental insights into biological processes. Particularly intriguing are genes in sequenced genomes that remain hypothetical and/or poorly represented in expressed sequence databases, and whose functions in health and disease remain unknown. Some of these are most probably implicated in organ-specific functions. Their characterization is essential to complete the annotation of sequenced genomes and is expected to contribute to advances in physiology and pathology. In order to achieve such goals, transcriptome studies on tissues rather than cultured cells, and eventually on a single cell type at a precise differentiation step are more likely to provide new information.

The epidermis is a highly specialized tissue mainly dedicated to the establishment of a barrier that restricts both water loss from the body and ingress of pathogens. The barrier function of the epidermis is known to involve the expression of numerous tissue-specific genes, most of which are specifically expressed in the late steps of keratinocyte differentiation. In order to establish and constantly maintain this barrier, keratinocytes undergo a complex, highly organized and tightly controlled differentiation program leading to cornification and finally to desquamation. During this process, cells migrate from the basal, proliferative layer to the surface, where they form the cornified layer (stratum corneum). According to the current model of skin epithelial maintenance, basal keratinocytes encompass a heterogeneous cell population that includes slow-cycling stem cells [1]. These stem cells give rise to transiently amplifying keratinocytes that constitute most of the basal layer. They divide only a few times and finally move upward while differentiating to form the spinous layer. The proliferating compartment is characterized by the specific expression of cell cycle regulators and integrin family members responsible for the attachment of the epidermis to the basement membrane. Growth arrested keratinocytes undergo differentiation, mainly characterized by a shift in cytokeratin expression from KRT5 (keratin 5) and KRT14 in the basal layer to KRT1 and KRT10 in suprabasal layers. As differentiation progresses, keratinocytes from the spinous layers progressively express a small number of specific differentiation markers, like involucrin. However, the differentiation program culminates in the granular layer, where keratinocytes express more than 30 epidermis-specific proteins, including proteins that are stored in cytosolic granules characteristic of granular keratinocytes (GKs). These proteins include well known components of the cornified layer, like loricrin and elafin, but also recently identified ones, such as keratinocyte differentiation associated protein (KDAP), hornerin, suprabasin, keratinocyte proline rich protein (hKPRP), and so on [2-5].

GKs undergo a special programmed cell death, called cornification, which gives rise to corneocytes that no longer exhibit transcriptional or translational activity and are devoid of organelles. Rather, their intracellullar content consists of a homogeneous matrix composed mainly of covalently linked keratins. The cornified envelope, a highly specialized insoluble structure, encapsulates corneocytes in place of their plasma membrane (see Kalinin *et al*. [6] for a recent review). The lipid-enriched extracellular matrix, which subserves the barrier, is produced by a highly active lipid factory mainly operative in the granular layer and comprises secretory organelles named the epidermal lamellar bodies [7]. In addition to the provision of lipids for the barrier, lamellar bodies deliver a large number of proteins, including lipid-processing enzymes, proteases and anti-proteases that regulate desquamation, antimicrobial peptides and corneodesmosin, an adhesive protein secondarily located in the external face of the desmosomes, as they turn into corneodesmosomes [8]. Therefore, the components of the stratum corneum, responsible for most of the protective cutaneous functions, are produced by GKs.

Transcriptome studies of selected cell types of the human epidermis are expected to contribute to the elucidation of the mechanisms responsible for barrier function. They will also shed further light on the causes of monogenic genodermatoses and the pathomechanisms of common complex skin disorders like psoriasis. However, present knowledge on the gene repertoire expressed by keratinocytes remains largely fragmentary. Among the approximately eight million human expressed sequence tags (ESTs) from the dbEST division of the GenBank database, only 1,210 are annotated as originating from the epidermis, although these are, in fact, derived from cultured keratinocytes, which do not fully recapitulate the complex *in vivo *differentiation program. In this article, we describe the results of a large-scale cDNA sequencing project on GKs of healthy human skin, purified by a new method. In order to characterize genes expressed at a low level and to avoid the repetitive sequencing of highly expressed ones, we used the ORESTES (open reading frame EST) method to prepare a large series of small size cDNA libraries using arbitrarily chosen primers for reverse transcription (RT) and PCR amplification [9]. The sequencing of about 25,000 clones has produced a list of 3,387 genes expressed by GKs. Some of them, analyzed by quantitative RT-PCR, were shown to be expressed in a cell-specific manner. This effort resulted in a large number of novel candidate genes of importance for the epidermis barrier function and the etiology of genodermatoses.

## Results

### Purification of human granular keratinocytes

**Figure 1:**
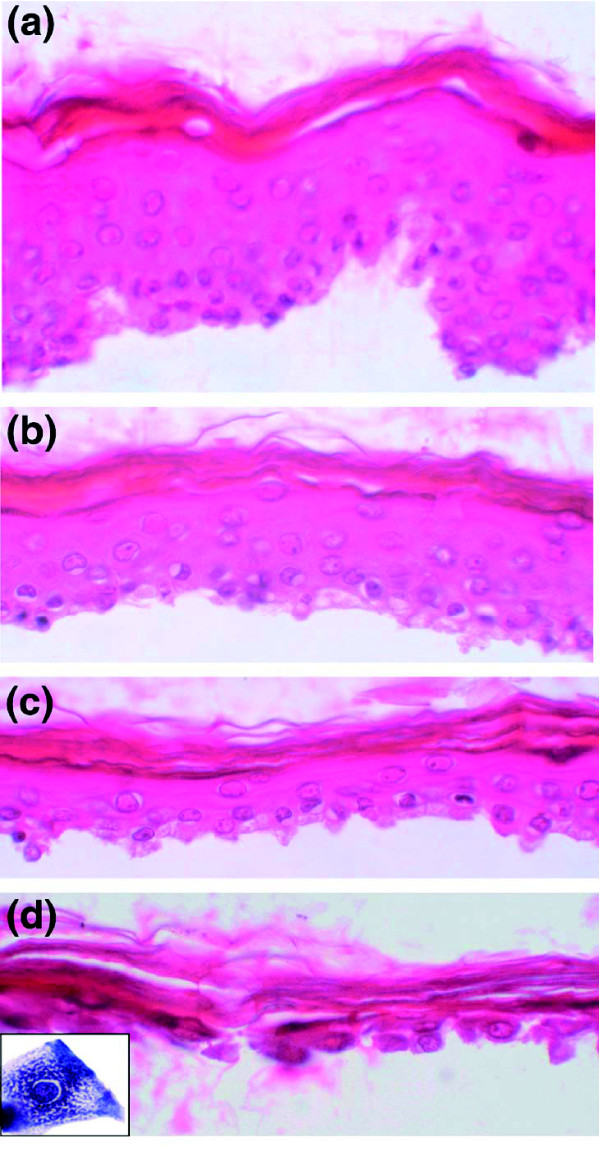
Histological analysis of epidermis samples. **(a) **Hematoxylin-eosin stained sections of entire epidermis after thermolysin incubation and removal of the dermis. **(b,c,d) **Epidermis fragments remaining after the first, second, and third trypsin incubation, respectively. Fragments shown in (d) are mainly composed of GKs attached to the cornified layer and constitute the T4 fraction. Inset: higher magnification showing the characteristic cytological aspect of a GK with cytoplasmic keratohyalin granules.

As a first step in this transcriptome project we devised a method to purify GKs. Iterative incubations of pieces of human epidermis with trypsin were performed to give three suspended cell fractions (hereafter named T1-T3) and finally to isolate cells attached to the stratum corneum (T4 fraction). Morphological analyses revealed that after three treatments, residual epidermal fragments were mostly composed of corneocytes and GKs (Figure 1). Quantitative real-time PCR was performed to quantify the enrichment in GKs. To first select a reference gene for normalization, the relative expression of eight housekeeping genes (*GAPDH*, *SOD1*, *ACTB*, *B2M*, *HPRT1*, *HMBS*, *TBP *and *UBC*) in each cell fraction (T1-T4) was analyzed using GeNorm [10]. In agreement with previous data [11], beta-2-microglobulin (*B2M*) appeared to be stably expressed during epidermis differentiation, and was thus chosen for normalization. In addition, we used the lectin Galectin-7 (*LSGAL7*), which was previously shown by *in situ *hybridization to be equally expressed in all epidermal layers [12]. *BPAG2 *(bullous pemphigoid antigen 2) or *KRT14*, and *KLK7 *(kallikrein 7, also called stratum corneum chymotryptic enzyme (*SCCE*)) were selected as specific for the basal layer or the GKs, respectively [13,14]. For four cell fractionations from different individuals, the mean T1/T4 expression ratio of *KRT14 *was 13, whereas the mean T4/T1 expression ratio of *SCCE *was approximately 130 (Table 1). The *KRT14 *ratio might be indicative of a slight contamination of the T4 fraction with basal keratinocytes. Nevertheless, the large *SCCE *ratio indicates that very few, if any, GKs were present in the T1 fraction. From this, we concluded that the T4 fraction was highly enriched in GKs and thus suitable for a large-scale study of their transcriptome.

**Table 1:** Expression ratios for *KRT14 *and *KLK7 *as measured by real-time PCR from four independent samples

	Sample no.			
	
Expression ratio	1	2	3	4
*KRT14 *(T1/T4)	7.5	5.9	25	13.6
*SCCE/KLK7 *(T4/T1)	164	189	120	54

### An ORESTES dataset from human granular keratinocytes

**Figure 2:**
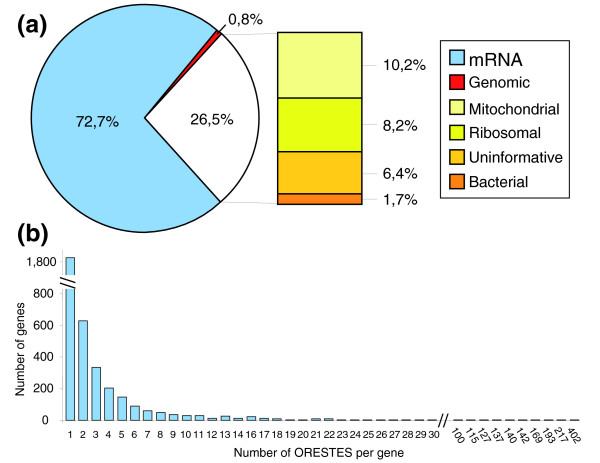
Analysis of the ORESTES dataset from GKs. **(a) **Pie graph of the 22,585 sequences obtained from the T4 fraction enriched in GKs. The treatment of the mRNA samples with DNAse resulted in minimal contamination with genomic sequences. Despite two rounds of polyA+ mRNA purification, rRNA sequences still represent approximately 8% of the dataset. **(b) **Histogram showing the number of ORESTES at each level of redundancy. The vast majority of genes are represented by less than five ORESTES, illustrating the normalization capability of that method. However, a small number of genes are represented by a large number of ORESTES (up to 402).

PolyA^+ ^RNA was extracted from the T4 fraction from individual 3 (Table 1) and used to generate cDNA mini-libraries using the ORESTES method [9]. This sample was chosen as it presents the highest T1/T4 expression ratio for the *KRT14 *gene, suggesting a low contamination of the T4 fraction by basal keratinocytes. This method uses arbitrarily chosen primers for reverse transcription and PCR amplification. The successful amplification of a mRNA thus depends primarily on partial sequence homology with the primer, rather than on its abundance. This, and the elimination of cDNA preparations that display prominent bands on gels (indicative of the selective amplification of particular mRNAs), results in a normalization process and allows the detection of rare transcripts. We constructed 150 cDNA libraries with different primers, the analysis of 100-200 clones from each leading to the production of 22,585 sequences (Figure 2a). Among these, 1,453 (approximately 6%) corresponded to empty plasmids or uninformative sequences, 377 (1.7%) were of bacterial origin, and 2,303 (10%) matched the human mitochondrial genome. Despite two rounds of polyA^+ ^RNA purification, 1,859 sequences (8.2%) arose from ribosomal RNA. In addition, 187 sequences corresponded to unspliced intergenic DNA and may reflect spurious transcriptional activity. The remaining 16,591 sequences (73%) matched known or predicted transcribed regions, of which 62% aligned with the human genome in several blocks, and thus corresponded to spliced transcripts. After clustering, we observed the transcription of 3,387 genes by GKs. Additionally, 23 sequences matched overlapping exons belonging to two genes transcribed in opposite orientations and thus could not be attributed to a single gene.

The normalization ability of the ORESTES method was examined by classifying genes according to the number of matching sequences in the dataset (Figure 2b). Half of the genes were represented by a unique sequence and 76.3% by three or less sequences, thus showing an acceptable level of normalization, with a mean of 4.6 ORESTES per gene. However, the ORESTES method only partially compensates for transcript abundance, as several genes were represented by a large ORESTES number. In these cases, we examined the number of sequences in the corresponding UniGene clusters, a rough measure of gene expression level. This revealed two situations: first, the gene is strongly expressed in many cell types including GKs (a high number of both ORESTES and UniGene entries); and second, the gene is particularly expressed in GKs (a high number of ORESTES, but low number of UniGene entries). The first category mainly includes housekeeping genes from the translation machinery (for example, *RPS8*, *EEF1A1*, *RPL3*, *RPL7A*, *RPL28*; Table 2). The second category contains genes previously described as implicated in epidermis barrier function (for example, *KRT1*, *DMKN*, *LEP7*, *FLG*, *KRT2A*, *SPRR2E*, *CASP14*, *CDSN*, *hKRP*, *SBSN*) and, interestingly, new candidates for this function (*TSPAN5*, *DUOX2*, *TMEM14C*, *SERPINA12*, *SLC22A5*, *FLG2*, *C7orf24*). Dermokine (*DMKN*), represented by 217 ORESTES, was shown to be selectively transcribed in mouse GKs by high-throughput *in situ *hybridization [15] and signal sequence trap [16] screens. The present ORESTES dataset allowed us to describe 13 novel human *DMKN *splicing isoforms with distinct subcellular locations and expression patterns [17].

**Table 2:** Representative sample of genes with the highest number of ORESTES

No. of ORESTES	Gene symbol	No. of UniGene ESTs	Full name (alias)
Ubiquitously expressed genes with a high number of UniGene ESTs			
142	*RPS8*	3,382	Ribosomal protein S8
115	*EEF1A1*	29,374	Eukaryotic translation elongation factor 1 alpha 1
77	*HLA-B*	4,536	Major histocompatibility complex, class I, B
71	*RPL3*	11,561	Ribosomal protein L3
62	*NCL*	2,970	Nucleolin
55	*RPL28*	2,394	Ribosomal protein L28
55	*RPL7A*	5,864	Ribosomal protein l7a
51	*RPSA*	5,623	Ribosomal protein SA
50	*PABPC1*	4,385	Poly(A) binding protein, cytoplasmic 1
34	*RPS18*	2,292	Ribosomal protein S18
			
Known epidermis specific genes			
402	*KRT1*	134	Keratin 1
217	*DMKN*	275	Dermokine
140	*LEP7*	5	Late envelope protein 7 (xp32)
100	*FLG*	5	Filaggrin
71	*KRT2A*	12	Keratin 2A
62	*SPRR2E*	36	Small proline-rich protein 2E
61	*CASP14*	19	Caspase 14
59	*CDSN*	91	Corneodesmosin
56	*hKPRP*	7	Human keratinocyte proline rich protein
54	*PKP1*	263	Plakophilin 1
32	*SBSN*	49	Suprabasin
30	*DSG1*	61	Desmoglein 1
			
Genes with unknown function			
193	*TSPAN5*	526	Tetraspanin 5
127	*DUOX2*	64	Dual oxidase 2
99	*TMEM14C*	476	Transmembrane protein 14C
99	*SERPINA12*	11	Serpin peptidase inhibitor, clade A, member 12
66	*SLC22A5*	142	Solute carrier family 22, member 5
56	*FLG2*	10	Filaggrin 2 (ifapsoriasin)
41	*C7orf24*	309	Chromosome 7 open reading frame 24

The ORESTES dataset was aligned with the human genome using BLAT [18]. The BLAT results were used to write a custom track that allows the visualization of the position of a particular ORESTE relative to other annotations such as RefSeq genes, vertebrate orthologues, single nucleotide polymorphisms, microarray expression data, and so on, and is freely available online [19]. A screen copy of a UCSC Genome Browser window showing the ORESTES obtained for the *C1orf81 *gene is presented as an example (Additional data file 1). Indeed, this gene was characterized and a cDNA (DQ983818) was cloned for the first time in this study (see below). Our dataset includes the 16,591 ESTs matching known or predicted transcribed regions. These sequences have also been deposited in public databases (GenBank:EL593304-EL595248, GenBank:CU442764-CU457374).

### Poorly represented genes in expressed sequence databases

As few sequencing projects from human epidermis have been performed so far (relative to other organs), genes expressed during the late steps of epidermis differentiation are poorly represented in sequence databases. Among the 3,375 genes from our set, 330 (10%) corresponded to UniGene clusters containing less than 100 mRNA/EST sequences, and were thus good candidates for epidermis late-expressed genes. These were subdivided into five classes. The first one contains all the genes (50) already known to be specifically expressed in the suprabasal layers (Table 3). This confirms that late-expressed genes are poorly represented in EST databases. The second class consisted of 31 genes with known or inferred functions that were previously known as mainly expressed in a specific tissue different from epidermis (Table 4). We suggest that some of them might play a specific role in epidermal differentiation. This could be the case for *SERPINA12*, *DUOX2*, and, to a lesser extent, *CASZ1*, which are represented by a large ORESTES number. We also suspect that *CLDN23 *might play an important role in GKs, since claudin-based tight junctions in the granular layer contribute to barrier function of the epidermis [20]. Accordingly, claudin-1-deficient mice display a lethal defect in skin permeability [21]. The third class gathered 32 uncharacterized paralogues of known genes (Table 5). The fourth class was composed of 105 genes that remain hypothetical and about which nothing is known regarding their normal function or disease relevance (Table 6). The fifth class contained genes that are expressed, most probably at low levels, in numerous tissues, but whose epidermal expression is, to the best of our knowledge, described here for the first time (Additional data file 2). Several genes from these five classes were selected to quantify their expression in the course of epidermal differentiation by real-time PCR (see below).

**Table 3:** Genes with less than 100 UniGene ESTs encoding known GK expressed proteins

No. of ORESTES	Gene symbol	No. of UniGene ESTs	Full name (alias)
2	*LCE1F*	1	Late cornified envelope 1F
4	*LCE2C*	1	Late cornified envelope 2C
1	*C1orf46*	2	Chromosome 1 open reading frame 46 (xp 33)
1	*LCE2A*	3	Late cornified envelope 2A
6	*LCE5A*	3	Late cornified envelope 5A
11	*LCE1A*	3	Late cornified envelope 1A
2	*PGLYRP3*	5	Peptidoglycan recognition protein 3
5	*LCE1C*	5	Late cornified envelope 1C
100	*FLG*	5	Filaggrin
140	*LEP7*	5	Late envelope protein 7
2	*RPTN*	6	Repetin
1	*LCE2B*	7	Late cornified envelope 2B
56	*hKPRP*	7	Human keratinocyte proline rich protein
9	*LOR*	11	Loricrin
71	*KRT2A*	12	Keratin 2A
2	*C1orf42*	15	Chromosome 1 open reading frame 42 (NICE-1)
5	*TGM5*	15	Transglutaminase 5
13	*DSC1*	17	Desmocollin 1
16	*KRT1B*	18	Keratin 1B
61	*CASP14*	19	Caspase 14
1	*CNFN*	20	Cornifelin
4	*CALML5*	25	Calmodulin-like 5
1	*ALOXE3*	28	Arachidonate lipoxygenase 3
8	*ALOX12B*	30	Arachidonate 12-lipoxygenase, 12R type
62	*SPRR2E*	36	Small proline-rich protein 2E
17	*IVL*	38	Involucrin
2	*EPPK1*	42	Epiplakin 1
5	*POU2F3*	45	POU domain, class 2, transcription factor 3 (oct-11)
4	*ICHTHYIN*	48	Ichthyin
32	*SBSN*	49	Suprabasin
2	*KLK8*	53	Kallikrein 8 (neuropsin/ovasin)
4	*TGM3*	54	Transglutaminase 3
1	*ABCA12*	55	ATP-binding cassette, sub-family A (ABC1), member 12
3	*PADI1*	56	Peptidylarginine deiminase, type I
30	*DSG1*	61	Desmoglein 1
2	*GJB3*	65	Gap junction protein, beta 3 (connexin 31)
1	*CALML3*	68	Calmodulin-like 3
13	*SASpase*	69	Skin aspartic protease
15	*KLK7/SCCE*	69	Kallikrein 7 (Stratum corneum chymotrypticenzyme)
6	*A2ML1*	76	Alpha-2-macroglobulin-like 1
1	*CST6*	78	Cystatin E/M
1	*SULT2B1*	80	Sulfotransferase family, cytosolic, 2B, member 1
2	*KLK11*	83	Kallikrein 11
3	*HAL*	86	Histidine ammonia-lyase (histidase)
14	*EVPL*	91	Envoplakin
59	*CDSN*	91	Corneodesmosin
3	*PDZK1IP1*	92	PDZK1 interacting protein 1
4	*TGM1*	92	Transglutaminase 1
2	*SERPINB8*	99	Serpin peptidase inhibitor, clade B, member 8
20	*SCEL*	99	Sciellin

**Table 4:** Genes with 100 or less UniGene ESTs, known as mainly expressed in a specific tissue different from epidermis

No. of ORESTES	Gene symbol	No. of UniGene ESTs	Full name	Main specificity
99	*SERPINA12*	11	Serpin peptidase inhibitor, clade A, member 12	Adipocytes
1	*BSND*	12	Bartter syndrome, infantile, with sensorineural deafness	Kidney and inner ear
1	*OPN1LW*	16	Opsin 1, long-wave-sensitive	Eye
5	*GRIN2*	16	G-protein-regulated inducer of neurite outgrowth	Brain
2	*IL1RL2*	24	Interleukin 1 receptor-like 2	Neurons
13	*LCTL*	25	Lactase-like	Kidney
1	*PPEF2*	31	Protein phosphatase, EF-hand calcium binding domain 2	Retina
2	*SLC6A3*	34	Solute carrier family 6, member 3	Neuron
3	*CDC42BPG*	41	CDC42 binding protein kinase gamma	Heart and skeletal muscle
4	*GPR75*	41	G protein-coupled receptor 75	Retina
1	*OTX1*	45	Orthodenticle homolog 1	Neurons
1	*K5B*	46	Keratin 5b	Tongue
1	*TBX15*	46	T-box 15	Embryo
3	*TMPRSS5*	53	Transmembrane protease, serine 5 (spinesin)	Spinal chord
3	*LEAP-2*	53	Liver-expressed antimicrobial peptide 2	Liver
1	*BMP8B*	56	Bone morphogenetic protein 8B	Embryo
1	*PTGFR*	59	Prostaglandin F receptor	Uterus
2	*TEC*	59	Tec protein tyrosine kinase	Hematopoietic cells
3	*SLC5A1*	60	Solute carrier family 5, member 1	Intestine and kidney
20	*CASZ1*	61	Castor homolog 1, zinc finger	Mesenchyme
2	*KCNJ12*	62	Potassium inwardly rectifying channel, subfamily J, 12	Heart
1	*P11*	64	26 serine protease	Placenta
14	*SERPINB7*	64	Serpin peptidase inhibitor, clade B, member 7	Mesangial cells
127	*DUOX2*	64	Dual oxidase 2	Thyroid
3	*GDPD2*	68	Glycerophosphodiester phosphodiesterase containing 2	Osteoblasts
3	*PDE11A*	75	Phosphodiesterase 11A	Testis
2	*CLDN23*	76	Claudin 23	Placenta
1	*PLCL4*	78	Phospholipase C-like 4	Neurons
1	*EYA4*	81	Eyes absent homolog 4	Heart and cochlea
1	*LIPH*	85	Lipase, member H	Intestine
1	*RBP3*	88	Retinol binding protein 3	Retina

**Table 5:** Genes with 100 or less UniGene ESTs, corresponding to uncharacterized paralogues of known genes

No. of ORESTES	Gene symbol	No. of UniGene ESTs	Full name
13	*ASAH3*	4	N-acylsphingosine amidohydrolase 3
1	*LIPL2 *(*LIPK*)	4	Lipase-like, ab-hydrolase domain containing 2
11	*CLEC2A*	7	C-type lectin domain family 2, member A
2	*IGFL3*	12	Insulin growth factor-like family member 3
3	*LYG2*	14	Lysozyme-like
1	*PNPLA1*	15	Patatin-like phospholipase domain containing 1
9	*GSDM1*	16	Gasdermin 1
1	*GRID2IP*	17	Glutamate receptor, ionotropic, delta 2 interacting protein
2	*IL1F7*	17	Interleukin 1 family, member 7
2	*FCRL6*	17	Fc receptor-like 6
3	*AADACL2*	20	Arylacetamide deacetylase-like 2
1	*LIPL3 *(*LIPM*)	20	Lipase-like, ab-hydrolase domain containing 3
1	*LAMB4*	26	Laminin, beta 4
10	*THEM5*	26	Thioesterase superfamily member 5
1	*FLJ90165*	27	Gamma-glutamyltransferase 6 homolog
3	*FLJ45651*	28	Phospholipase A2, group IVE
1	*LPIN3*	31	Lipin 3
1	*SLC25A34*	36	Solute carrier family 25, member 34
1	*GPR115*	37	G protein-coupled receptor 115
1	*LRP5L*	41	Low density lipoprotein receptor-related protein 5-like
1	*HSPC105*	45	NAD(P) dependent steroid dehydrogenase-like
1	*QPCTL*	52	Glutaminyl-peptide cyclotransferase-like
3	*PLA2G4F*	56	Phospholipase A2, group IVF
1	*KIAA0605*	58	ADAMTS-like 2
1	*CTGLF1*	60	Centaurin, gamma-like family, member 1
2	*UGT3A2*	65	UDP glycosyltransferase 3 family, polypeptide A2
1	*GALNT17*	74	Polypeptide N-acetylgalactosaminyltransferase 17
1	*BAIAP2L2*	76	BAI1-associated protein 2-like 2
1	*FLJ43692*	80	ARHGEF5-like
1	*VILL*	86	Villin-like
1	*LOC203427*	87	Similar to solute carrier family 25, member 16
1	*IL17RE*	100	Interleukin 17 receptor E

**Table 6:** Unknown genes with 100 or less UniGene ESTs

No. of ORESTES	Gene symbol	No. of UniGene ESTs	Full name
1	*FLJ43861*	3	Flj43861
1	*LOC389791*	3	Hypothetical gene supported by AK094537
1	*LOC285435*	4	Hypothetical LOC285435
2	*LOC387846*	6	Hypothetical LOC387846
4	*LOC401062*	6	Hypothetical gene supported by AK092973
1	*IMAGE:5260914*	7	Image:5260914
5	*LOC338667*	7	Hypothetical protein LOC338667
5	*PSORS1C2*	8	Psoriasis susceptibility 1 candidate 2
1	*DKFZp779B1540*	9	Hypothetical protein dkfzp779b1540
5	*C14orf72*	9	Chromosome 14 open reading frame 72
1	*FLJ37989*	10	Flj37989
56	*FLG2*	10	Filaggrin 2 (ifapsoriasin)
10	*WFDC5*	11	WAP four-disulfide core domain 5
1	*LOC402110*	12	Hypothetical LOC402110
1	*PLEKHN1*	12	Pleckstrin homology domain containing, family N member 1
1	*LOC441240*	13	Hypothetical protein LOC441240
4	*FLJ38159*	14	Hypothetical protein FLJ38159
1	*C1orf177*	15	Chromosome 1 open reading frame 177
1	*HMCN2*	16	Hemicentin 2
2	*MGC23985*	16	Similar to AVLV472
1	*OFCC1*	17	Orofacial cleft 1 candidate 1
1	*LOC441860*	17	Novel KRAB box containing C2H2 type zinc finger protein
5	*AMIGO3*	18	Adhesion molecule with Ig-like domain 3
1	*LOC441257*	20	Hypothetical protein LOC441257
1	*LOC285484*	20	Hypothetical protein LOC285484
1	*C20orf91*	20	Chromosome 20 open reading frame 91
1	*LOC202460*	21	Hypothetical protein LOC202460
2	*FLJ25664*	21	Flj25664
8	*FLJ41623*	21	Flj41623
10	*LOC342897*	21	Similar to F-box only protein 2
1	*LOC339237*	23	Similar to Envoplakin
13	*LOC126248*	24	Hypothetical protein LOC126248
1	*LOC389142*	27	Hypothetical LOC389142
3	*C20orf95*	28	Chromosome 20 open reading frame 95
1	*FKBP9L*	31	FK506 binding protein 9-like
1	*FNDC8*	31	Fibronectin type III domain containing 8
3	*FLJ46311*	31	FLJ46311 protein
1	*C3orf47*	33	Chromosome 3 open reading frame 47
1	*LOC283143*	35	Hypothetical protein LOC283143
1	*LOC388727*	35	Hypothetical LOC388727
2	*FLJ44317*	35	Flj44317
1	*FLJ31184*	36	Flj31184
1	*LOC125893*	39	Hypothetical protein LOC125893
1	*ZNF311*	40	Zinc finger protein 311
1	*BC041923*	40	Image:5300199
2	*ZNF600*	43	Zinc finger protein 600
3	*MCMDC1*	43	Minichromosome maintenance deficient domain containing 1
3	*FLJ13646*	46	Hypothetical protein FLJ13646
1	*C14orf121*	48	Chromosome 14 open reading frame 121
1	*FAM83F*	49	Family with sequence similarity 83, member F
3	*ABHD9*	51	Abhydrolase domain containing 9
1	*LOC134466*	52	Hypothetical protein LOC134466
1	*CXorf33*	52	Chromosome X open reading frame 33
2	*FLJ25006*	52	Hypothetical protein FLJ25006
2	*DKFZp434N062*	53	Hypothetical protein dkfzp434n062
9	*LASS3*	53	LAG1 longevity assurance homolog 3
1	*C14orf21*	54	Chromosome 14 open reading frame 21
1	*C17orf67*	56	Chromosome 17 open reading frame 67
1	*FAM62C*	58	Family with sequence similarity 62, member C
2	*C14orf29*	60	Chromosome 14 open reading frame 29
1	*FLJ21736*	61	Esterase 31
3	*LOC349114*	61	Hypothetical protein LOC349114
1	*MGC26885*	62	Hypothetical protein MGC26885
1	*SMA3*	62	Sma3
8	*FAM46B*	62	Family with sequence similarity 46, member B
13	*ELMOD1*	62	ELMO/CED-12 domain containing 1
3	*DENND2C*	63	DENN/MADD domain containing 2C
13	*ANKRD35*	64	Ankyrin repeat domain 35
5	*LOC401553*	66	Hypothetical gene supported by BC019073
1	*LOC390927*	67	Similar to zinc finger protein 569
1	*ZNF696*	67	Zinc finger protein 696
2	*CCDC9*	69	Coiled-coil domain containing 9
6	*C15orf40*	70	Chromosome 15 open reading frame 40
1	*LOC148137*	73	Hypothetical protein BC017947
1	*ZC3H12C*	74	Zinc finger CCCH-type containing 12C
1	*APXL2*	74	Apical protein 2
1	*ZMYND19*	75	Zinc finger, MYND-type containing 19
1	*LRRC37B*	77	Leucine rich repeat containing 37B
2	*FLJ32356*	77	Family with sequence similarity 109, member A
3	*DQX1*	77	DEAQ box polypeptide 1
2	*C9orf9*	79	Chromosome 9 open reading frame 9
4	*FNDC6*	79	Fibronectin type III domain containing 6
1	*MSTP9*	82	Macrophage stimulating, pseudogene 9
1	*HES2*	83	Hairy and enhancer of split 2
1	*FLJ37464*	84	Hypothetical protein FLJ37464
1	*KIAA1862*	84	KIAA1862 protein
1	*LOC196264*	86	Hypothetical protein LOC196264
1	*C1orf51*	86	Chromosome 1 open reading frame 51
2	*ANKRD5*	86	Ankyrin repeat domain 5
1	*CXorf23*	87	Chromosome X open reading frame 23
2	*SMCR8*	88	Smith-Magenis syndrome chromosome region, candidate 8
2	*DKFZp686L1814*	88	Hypothetical protein dkfzp686l1814
2	*MGC34647*	88	Hypothetical protein MGC34647
1	*C6orf105*	89	Chromosome 6 open reading frame 105
1	*FLJ23186*	90	Chromosome 3 open reading frame 52
1	*SAMD10*	91	Sterile alpha motif domain containing 10
2	*KIAA1287*	91	Kiaa1287
2	*C19orf36*	91	Chromosome 19 open reading frame 36
1	*ZNF662*	93	Zinc finger protein 662
2	*ZNF429*	97	Zinc finger protein 429
12	*SMPD3*	98	Sphingomyelin phosphodiesterase 3
1	*PCGF1*	100	Polycomb group ring finger 1
1	*C17orf61*	100	Chromosome 17 open reading frame 61
1	*KIAA1853*	100	Kiaa1853
1	*C22orf23*	100	Chromosome 22 open reading frame 23

### Expressed retrogenes and pseudogenes

Pseudogenes generally correspond to retrocopies with many disruptions in their open reading frame (ORF). However, it is now recognized that a large number of retrocopies are transcribed and can encode functional proteins [22]. Among the top 50 transcribed retrocopies reported by these authors, 11 were detected in GKs by the ORESTES method. Among these, calmodulin-like 3 (*CALML3*) was previously shown to be specific for keratinocyte terminal differentiation [23]. We identified two other expressed retrogenes corresponding to the retrotransposition of the cutaneous T-cell lymphoma associated antigen 5 (*CTAGE5*), and CCR4-NOT transcription complex, subunit 6-like (*CNOT6L*). These genes can be considered as 'intact', that is, they show no disablements such as premature stop codons or frameshift mutations when compared to the ORF of their parental genes. Of note, the *CNOT6L *retrogene is specific for hominoids (Additional data file 3), while the *CTAGE5 *retrogene is specific for primates (data not shown).

Moreover, six unspliced ORESTES correspond to a part of intron 8 of the *PPP2R5A *gene, and include the small nucleolar RNA (snoRNA) U98b sequence. The snoRNAs are non-protein-coding RNAs that guide the 2'O-ribose methylation (C/D box snoRNAs) or the pseudouridylation (H/ACA box snoRNAs) of ribosomal RNAs, and are generally processed from introns of RNA polymerase II transcripts [24]. Interestingly, the U98b snoRNA is a primate-specific retroposon of the *ACA16 *snoRNA hosted by the *PNAS-123 *gene [25]. We thus suggest that the ORESTES from the *PPP2R5A *gene correspond to a precursor form of the U98b snoRNA, and that snoRNA retroposons can indeed be expressed when located in an intron of a new host gene in the sense orientation. Therefore, our ORESTES dataset included transcripts from retrogenes, originating either from spliced pre-mRNAs or from an intron-encoded snoRNA gene.

### Non-protein-coding genes

We obtained two long spliced ORESTES highly similar to the BC070486 mRNA form of the *GAS5 *gene, a non-protein-coding gene that belongs to the 'growth arrest specific' family but is disrupted in its ORF by a premature stop codon. The *GAS5 *gene is the host gene for 10 C/D box snoRNAs [26]. Other snoRNA host genes included in our ORESTES dataset are *RPS11*, *RPS12*, *RPL10 *and *EIF4A1*. In certain cases, ORESTES contain the snoRNA sequence (U39B in *RPS11*, mgU6-77 in *EIF4A1*, U70 in *RPL10*), and probably correspond to alternative splicing forms of the host gene mRNA, with intron retention.

We furthermore obtained sequences for long, non-protein-coding transcripts. Metastasis associated lung adenocarcinoma transcript 1, (*MALAT-1*, 22 ORESTES) is a conserved long non-protein-coding RNA (>8,000 nucleotides (nt)) of unknown function that is highly expressed in numerous healthy organs and overexpressed in metastatic non-small cell lung carcinomas [27]. Close to *MALAT-1 *on 11q13.1, trophoblast-derived noncoding RNA (*TncRNA*, 44 ORESTES) is a 481 nucleotide (nt), non-protein-coding RNA involved in trophoblastic major histocompatibility complex suppression by inhibiting class II transactivator (*CIITA*) transcription [28]. *H19 *is a non-protein-coding, maternally imprinted mRNA (two spliced ORESTES) [29] that is highly transcribed in extraembryonic and fetal tissues, as well as in adult skeletal muscle. It has been shown that *H19 *is involved in the genomic imprinting of the insulin-like growth factor 2 (*IGF2*) gene [30]. Moreover, *IGF2 *is expressed throughout the epidermis [31] and its overexpression increases the thickness of the epidermis and the proportion of dividing cells in the basal layer [32]. We suggest that *H19 *could participate in the regulation of *IGF2 *transcription by maintaining the genomic imprinting of its promoter in adult epidermis. In addition to numerous protein-coding genes, we thus detected several non-protein-coding RNAs whose expression in the epidermis had not been previously assessed, evoking the possibility that they might play a specific role in this tissue.

### Real-time PCR expression profiling of selected genes

Genes involved in the establishment of the skin barrier are expected to be specifically overexpressed by granular keratinocytes. To compare the expression levels of candidate genes between the basal layer and GKs, quantitative real-time PCR experiments were performed with the T4 and T1 cell fractions. Based on predicted domains and homologies, 73 genes represented by less than 100 ESTs were selected (Table 7). The relative T4/T1 ratio could not be calculated for 20 of them due to very low expression levels. Ten genes were equally expressed in the two layers, and nine were overexpressed in the basal layer, even if expressed at a low level in the granular layer. Interestingly, 33 were overexpressed in the granular layer with T4/T1 ratios ranging from 6 to 800. For several genes, the T4/T1 expression ratio was thus much larger than that observed for the *KLK7 *gene, used as a specific marker of the GKs in our cell purification experiments (Table 1). Therefore, these data emphasize the high degree of purity of the GKs we have purified from healthy human skin. They also provide one with new, highly specific markers for this cell type.

**Table 7:** Comparison of gene expression in the T4 and T1 cell fractions by real-time PCR

No. of ORESTES	Gene symbol	Full name	No. of UniGene ESTs	T4/T1 expression ratio
56	*FLG2*	Filaggrin 2 (ifapsoriasin)	10	800
9	*GSDM1*	Gasdermin 1	16	800
13	*ASAH3*	N-acylsphingosine amidohydrolase 3	4	300
2	*IL1F7*	Interleukin 1 family, member 7	17	200
13	*ELMOD1*	ELMO/CED-12 domain containing 1	62	160
0	*LIPL4 *(*LIPN*)	Lipase-like, ab-hydrolase domain containing 4	9	150
1	*LIPL3 *(*LIPM*)	Lipase-like, ab-hydrolase domain containing 3	20	130
3	*C20orf95*	Chromosome 20 open reading frame 95	28	120
1	*WFDC12*	WAP four-disulfide core domain 12 (WAP2)	3	110
1	*LIPL2 *(*LIPK*)	Lipase-like, ab-hydrolase domain containing 2	4	100
12	*SMPD3*	Sphingomyelin phosphodiesterase 3	98	70
10	*LOC440449*	Similar to WDNM1 homolog (LOC645638)	-	50
99	*SERPINA12*	Serpin peptidase inhibitor, clade A, member 12	11	50
1	*P11*	26 serine protease	64	35
5	*PSORS1C2*	Psoriasis susceptibility 1 candidate 2	8	30
1	*C3orf52*	Chromosome 3 open reading frame 52	90	30
2	*CLDN23*	Claudin 23	76	25
1	*PNPLA1*	Patatin-like phospholipase domain containing 1	15	20
10	*THEM5*	Thioesterase superfamily member 5	26	20
3	*ABHD9*	Abhydrolase domain containing 9	51	20
2	*TMEM16H*	Transmembrane protein 16H	78	16
6	*SERPINB12*	Serpin peptidase inhibitor, clade B, member 12	6	15
1	*PLEKHN1*	Pleckstrin homology domain containing, family N member 1	12	12
1	*FAM83F*	Family with sequence similarity 83, member F	49	12
3	*AADACL2*	Arylacetamide deacetylase-like 2	20	12
10	*LOC342897*	Similar to F-box only protein 2	21	10
9	*LASS3*	LAG1 longevity assurance homolog 3	53	10
20	*CASZ1*	Castor homolog 1, zinc finger	61	10
8	*FAM46B*	Family with sequence similarity 46, member B	62	10
14	*SERPINB7*	Serpin peptidase inhibitor, clade B, member 7	64	10
1	*CARD14*	Caspase recruitment domain family, member 14	86	8
1	*GGT6*	Gamma-glutamyltransferase 6 homolog	27	6
1	*CXorf33*	Chromosome X open reading frame 33	52	6
10	*WFDC5*	WAP four-disulfide core domain 5 (WAP1)	11	1
5	*KIAA0514*	Kiaa0514	16	1
5	*AMIGO3*	Adhesion molecule with Ig-like domain 3	18	1
3	*PLA2G4E*	Phospholipase A2, group IVE	28	1
1	*HSPC105*	NAD(P) dependent steroid dehydrogenase-like	45	1
3	*PLA2G4F*	Phospholipase A2, group IVF	56	1
127	*DUOX2*	Dual oxidase 2	64	1
1	*APXL2*	Apical protein 2	74	1
3	*RAB38*	RAB38, member RAS oncogene family	79	1
1	*AFMID*	Arylformamidase	87	1
2	*ANKRD5*	Ankyrin repeat domain 5	86	0.46
3	*MCMDC1*	Minichromosome maintenance deficient domain containing 1	43	0.4
1	*C14orf21*	Chromosome 14 open reading frame 21	54	0.38
3	*LEAP-2*	Liver-expressed antimicrobial peptide 2	53	0.36
1	*LRP5L*	Low density lipoprotein receptor-related protein 5-like	41	0.35
4	*FNDC6*	Fibronectin type III domain containing 6	79	0.3
2	*KIAA1287*	Kiaa1287	91	0.28

Not determined: *FLJ21736*, *HES2*, *ZNF696*, *LYG2*, *MGC34829*, *KIAA0963*, *ALKBH*, *ADAMTSL2*, *c6orf105*, *AW367250*, *KCNJ12*, *LOC338667*, *TMPRSS5*, *CLEC2A*, *FLJ21736*, *c14orf29*, *c14orf72*, *KRBA1*, *FLJ37464*, *LOC134466*.

### Identification of new genes

#### *FLG2*

**Figure 3:**
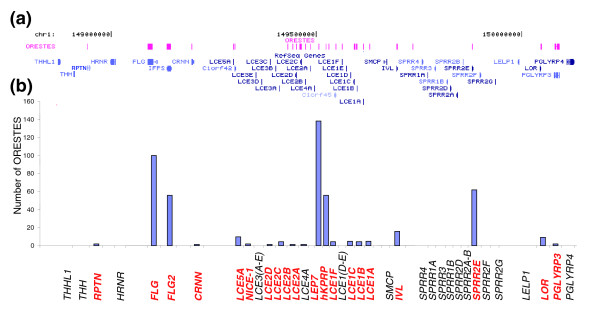
Genes of the EDC present in the ORESTES dataset. **(a) **Screen copy of a UCSC Genome Browser window (chr1:150,300,000-151,590,000; hg17, May 2004) showing the RefSeq genes from the EDC, and the ORESTE custom track. **(b) **Number of ORESTES for each gene of the locus. The genes for which at least one ORESTE was sequenced are in red bold characters.

**Figure 4:**
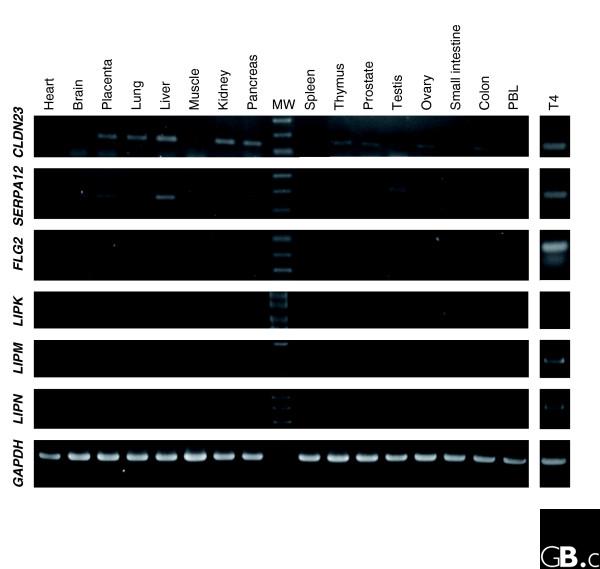
Expression profile of newly identified genes. PCR experiments were performed with a commercial panel of cDNAs from 16 human tissues (PBL, peripheral blood leukocytes) and with cDNAs prepared from the T4 fraction enriched in GKs. For each gene, PCR primers were chosen to amplify a cDNA fragment encompassing at least two exons. Note the highly specific expression pattern of *FLG2*, *LIPK*, *M*, *N*, and, to a lesser extent, *SERPINA12 *genes. The apparent size variation of the *CLDN23 *fragment results from an artifactual gel distortion. Expression of *GAPDH*, assessed with the primers provided by the manufacturer, was used as a control.

The epidermal differentiation complex (EDC) spans 1.62 megabases on 1q21.3 and contains approximately 50 genes specifically involved in the barrier function, such as those encoding involucrin, loricrin, filaggrin, small proline rich proteins (*SPRR1-4*) or late cornified envelope proteins (*LCE1-5*) (Figure 3a). We cloned many sequences corresponding to known genes of this locus (Figure 3b), but also a large number of sequences for a previously poorly characterized transcript encoding filaggrin 2 (*FLG2*; also called ifapsoriasin (*IFPS*); (GenBank:AY827490)). *FLG2 *displays features of the fused-family genes (encoding filaggrin, trichohyalin, or repetin), with three exons and a large predicted protein sequence (2,391 amino acids) containing two calcium binding EF-hand domains and a large domain made of repeated segments of about 25 amino acids. The amino acid composition of *FLG2 *is very similar to that of filaggrin, with a high content of serine (22%), glycine (20%), histidine (10%) and glutamine (10%). The expression of this gene is likely restricted to the epidermis, as shown by PCR on a panel of cDNAs from 16 healthy human tissues and organs (Figure 4). Real-time PCR also showed a strong overexpression of the *FLG2 *gene in GKs, with a T4/T1 ratio of 800 (Table 7). These results thus suggest that this gene is a new functional member of the EDC complex, in agreement with its similarity to the filaggrin gene, whose function in the epidermal barrier is well established.

#### Lipase-like genes

**Figure 5:**
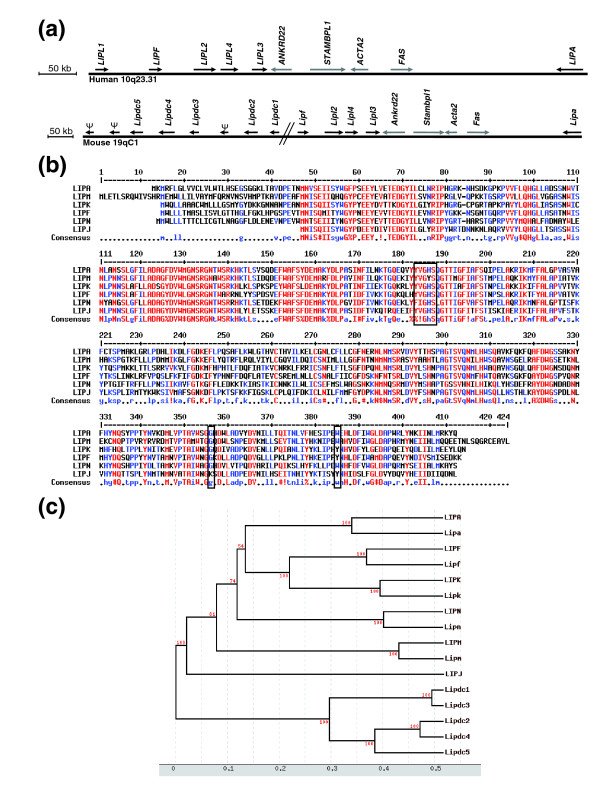
Analysis of new human lipase genes. **(a) **Schematic representation of the lipase gene cluster on chromosome 10q23.31. Six lipase genes, including the newly described *LIPL1 *(*LIPJ*), *LIPL2 *(*LIPK*), *LIPL3 *(*LIPM*) and *LIPL4 *(*LIPN*), form a cluster also containing four unrelated genes. **(b) **Alignment of the protein sequences of the six human lipases from the chromosome 10q23.31 cluster. The amino acids of the catalytic triad are boxed. The alignment was generated with Multalin software [71]. **(c) **Hierarchical clustering of human and mouse abhydro-lipase gene family members. The human LIPA, LIPF, LIPK, LIPM and LIPN, but not LIPJ, proteins have clear mouse orthologues (lower-case gene names). The six hypothetical mouse genes found in place of the *LIPJ *gene (*Lipdc1-5*) form a separate branch of the phylogenetic tree. This tree was generated with the Tree Top software [72]. Bootstrap values are indicated in red.

Two ORESTES were identified as the human orthologues of the murine lipases *Lipl2 *(NM_172837) and *Lipl3 *(BC031933), previously identified by large-scale mouse cDNA sequencing by the Riken Institute [33] and the Mammalian Gene Collection program [34], respectively. The corresponding human genes *LIPL2 *and *LIPL3 *were clustered in a 665 kB interval on chromosome 10q23.31 with genes encoding two experimentally characterized lipases, *LIPA *(lysosomal acid lipase, MIM +27,8000) and *LIPF *(gastric lipase, MIM #601980) and two hypothetical lipase-like proteins, *LIPL1 *and *LIPL4 *(Figure 5a). Therefore, our study contributed to the elucidation of a specialized human genomic locus that includes six lipase genes and four other genes (*ANKRD22*, *STAMBPL1*, *ACT2 *and *FAS*) of apparently unrelated function (Figure 5a). In accordance with the Hugo Gene Nomenclature Committee (HGNC), these new hypothetical lipase genes, *LIPL1*, *LIPL2*, *LIPL3 *and *LIPL4*, have been renamed *LIPJ*, *LIPK*, *LIPM*, and *LIPN*, respectively.

Rapid amplification of cDNA ends (RACE) cloning experiments of the human *LIPK *mRNA localized the cap site 165 nt upstream of the conceptual ATG initiation codon. For the *LIPM *and *LIPN *mRNAs, the entire coding sequences have been cloned using primers deduced from alignments with the mouse cDNAs (GenBank:EF426482, GenBank:EF426484 and GenBank:EF426483).

The conceptual translation of *LIPJ*, *LIPK*, *LIPM*, and *LIPN *ORFs led to proteins of 366, 399, 423 and 398 amino acids, respectively. As is the case for LIPA and LIPF, these proteins contain an abhydrolase associated lipase region (PFAM PF04083) with an alpha/beta hydrolase fold domain (PFAM PF00561). More precisely, they display the characteristic Ser-Asp-His catalytic triad (Figure 5b) also present in various hydrolases, such as serine proteases [35,36]. Surprisingly, LIPJ (NM_001010939) appeared as the only member in this family to lack a cleavable signal peptide (Figure 5b) and might thus play a distinct role in human physiology.

The clustering of six lipase genes on the human 10q23.31 locus led us to examine in detail the syntenic mouse locus (19qC1), where the orthologous genes except for *LIPN *and *LIPJ *had already been identified or predicted,. Sequence analysis showed that the AK154333 cDNA clearly corresponds to the *LIPN *orthologue (89% homology). As in the human genome, this gene resides between the *Lipl2 *and *Lipl3 *genes. Intriguingly, we could not precisely identify a single orthologue of *LIPJ*. Instead, we found upstream of the *Lipf *gene a cluster of five predicted genes encoding secreted alpha/beta hydrolases as well as at least three pseudogenes containing premature stop codons (Figure 5a). A syntenic cluster, albeit of smaller size, resides in the rat genome. These five hypothetical functional mouse lipases form by themselves a separate group in the phylogenic tree (Figure 5c; Additional data file 4). Their genomic localization suggests that they could have arisen from a large expansion by tandem duplications well after the separation of the rodents from the primate ancestors, or from a lipase gene lost in the human genome. These five new genes have thus been renamed *Lipdc1-5 *for lipase domain containing1-5 in accordance with the Mouse Gene Nomenclature Committee (MGNC) (Additional data file 5).

The human *LIPK*, *LIPM *and *LIPN *genes appear to be exclusively expressed in the epidermis, as shown by PCR on a panel of 16 cDNAs (Figure 4). Their expression was highly specific for GKs, with real-time PCR T4/T1 ratios of 100-150 (Table 7). *LIPJ *expression was also detected in the epidermis by PCR, but was too weak to allow real-time PCR experiments to be performed. These results strongly suggest that the *LIPK*, *LIPM *and *LIPN *genes play a highly specific role in the last step of keratinocyte differentiation. Although highly related, the LIPJ protein might play a different role, as previously suggested by its lack of a signal peptide.

#### *C1orf81*

**Figure 6:**
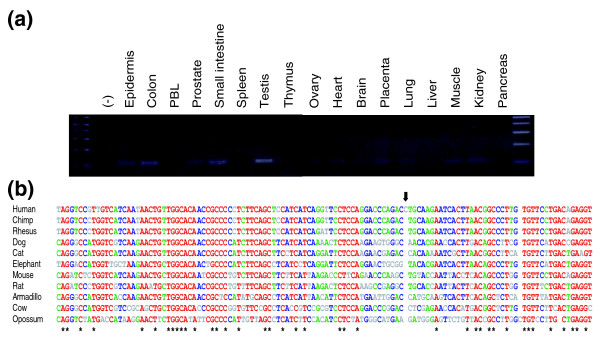
*C1orf81 *mRNA expression and conservation of the eighth exon among mammals. **(a) **Expression pattern of the *C1orf81 *gene. PCR was performed with a commercial panel of cDNAs from 16 human tissues (PBL, peripheral blood leukocytes) and with cDNA prepared from epidermis. The amplified fragment (120 nt) encompasses exons 13-14. **(b) **Sequence alignment of the eighth exon of the *C1orf81 *gene from 11 mammals. The sequences were retrieved from the multiz17way table of the UCSC Genome Browser [73], and from a BLAST search of the cat genome. The consensus splicing signals are boxed. The black arrow indicates the single nucleotide insertion in the human gene. The alignment was created with Multalin software [71]. Asterisks indicate the positions conserved in all the sequences. The colors correspond to various levels of consensus, with red for high consensus and grey for low consensus.

Six spliced ORESTES corresponded to a totally unknown gene without mRNA sequences in the databases. We performed RACE experiments and identified 16 exons and 15 introns with canonical splice sites and a consensus polyadenylation signal (GenBank:DQ983818). In agreement with the HGNC, we named this gene *C1orf81*. To date, only 16 ESTs are present in the databases, one from testis and the others from pooled tissues (Additional data file 1). PCR on a panel of cDNAs from 16 healthy human tissues and organs showed that spliced transcripts for this gene are detectable in most samples (Figure 6a). From its conceptual translation, the *C1orf81 *mRNA might encode a 373 amino acid protein. However, a second ORF was present in another frame that overlapped the first one by 87 amino acids. This suggested that a longer protein might be produced by a ribosomal frameshift mechanism. We explored the *C1orf81 *gene orthologues in vertebrate genomes, and particularly the region corresponding to the ORF overlap (exons 7-9). Compared with the *C1orf81 *gene from 10 other mammals, exon 8 of the human gene contained a 1 base-pair insertion that creates the frameshift (Figure 6b). The possibility of a sequence error in the present human genome assembly was excluded by sequencing the exon 8 region from two individuals. Accordingly, the human *C1orf81 *gene product would be 373 amino acids long, but 762 and 714 amino acids long in the chimpanzee and rhesus monkey, respectively. In all cases, the analysis of the peptide sequence did not reveal known domains or signal peptide. Further studies are needed to uncover the role of this new gene in mammalian physiology, and evaluate the consequences of its possible inactivation in human.

## Discussion

We have described here the first large-scale study of the transcriptome of human epidermal cells. As we are interested in genes that participate in the barrier function of the skin, we focused on GKs. They correspond to the ultimate step in the course of keratinocyte differentiation, and are the last epidermal cells to display gene expression activity before undergoing a particular programmed cell death leading to cornification. Because these cells represent less than 10% of the epidermis population, a preliminary step was to design an efficient purification method starting from healthy human skin fragments. After unsuccessful attempts using size filtration, Ficoll gradients, and fluorescence-activated cell sorting, we used successive short-term enzyme incubations to progressively detach cells from the deep layers, and purify the cells that remain attached to the cornified layer. These incubations were performed at 4°C to stop cellular metabolic activity and preserve the mRNA pool from degradation. This point is highly relevant as many growth factors, cell cycle regulators and transcription factors are encoded by short-lived mRNA. Quantitative PCR experiments were used to assess the relative expression levels of several genes in the successive cell fractions.

### ORESTE technique

In the present transcriptome project, the ORESTE methodology was selected because it produces sequences that are distributed predominantly within the central part of the corresponding transcripts and is biased towards less-abundant mRNAs [9]. When using arbitrarily chosen primers for reverse transcription and PCR, the amplification of a given transcript is proportional to its length and to the probability for the primer to anneal at low stringency (37°C). This conceptual normalization was secondarily strengthened by removing cDNA libraries with highly abundant amplification products. After the elimination of irrelevant sequences, we produced 16,591 ORESTES representing 3,387 genes. The distribution of the ORESTE number per gene (Figure 2) and the low number of ESTs in the corresponding UniGene entries fully confirmed that the transcript normalization obtained with this method is at least comparable to that of hybridization-subtraction techniques (for an example, see [37]).

### Overrepresented genes

Nevertheless, a few genes were represented by more than 100 sequences obtained from different mini-libraries. These include ubiquitous, highly expressed genes like *RPS8 *and *EEF1A1*, and genes already known to be highly transcribed in GKs (those encoding keratin 1, late envelope protein 7 or profilaggrin). Other members of this class, *DMKN *(formerly *ZD52F10*), *SERPINA12 *and *FLG2 *were not previously known to be overexpressed in the skin, and were thus studied in more detail. Two isoforms of transcripts for the dermokine gene were previously reported [15,16] and we describe 13 novel mRNA isoforms that are either ubiquitous or epidermis-specific depending upon alternative promoter usage. The epidermis-specific forms encode secreted proteins of still unclear function, and are abundantly transcribed in this tissue [17].

*SERPINA12*, also named *Vaspin *(visceral adipose tissue-derived serine protease inhibitor), is an extracellular serine protease inhibitor that displays insulin-sensitizing effects [38]. In addition to the previously documented expression in the liver, we observed that the *SERPINA12 *gene is highly expressed in the human epidermis (Figure 4). Moreover, real-time PCR showed that it is specifically expressed by GKs (T4/T1 ratio of 50). The Serpin A12 protein might thus play a role in the regulation of the complex balance between various proteases and their inhibitors operative in the desquamation process.

The filaggrin 2 (*FLG2*; or ifapsoriasin (*IFPS*)) gene is a new member of the EDC (chromosome 1q21.3), a cluster of approximately 50 genes involved in cornification. Our real-time RT-PCR experiments revealed that *FLG2 *expression displays strong specificity for GK. Interestingly, its composition is very close to that of filaggrin. The degradation of filaggrin is considered to be at the origin of the free amino acid pool of the natural moisturizing factor, which is capable of attracting and retaining water in the cornified layer to achieve skin softness and flexibility. We suggest that FLG2 might have a similar function.

### *C1orf81 *might have lost its function during hominization

A new gene characterized in this study, *C1orf81*, is particularly intriguing as the corresponding transcript (2,284 nt) displays an ORF disruption by a human-specific, single nucleotide insertion in the eighth exon (Figure 6b). As a result, a premature stop codon resides in exon 9, 103 nt upstream of its 3' end. Consequently, the human *C1orf81 *mRNA might be degraded by the nonsense mediated mRNA decay (NMD) pathway [39]. This appears unlikely, however, as its expression in various tissues was detected by RT-PCR (Figure 6a). However, its expression in epidermis was too weak to perform real-time PCR experiments. Our analysis suggests that the human *C1orf81 *gene encodes a truncated protein relative to other mammalian species, including the rhesus monkey. Nevertheless, we do not exclude the possibility that the translation of the human *C1orf81 *mRNA might produce a full-length protein by a +1 ribosomal frameshift mechanism. To our knowledge, such a phenomenon has been uniquely described in human for the ornithine decarboxylase antizyme gene family [40]. This would thus constitute a further example of a (partial) loss of gene function during hominoid evolution. The systematic comparison of the human and chimpanzee genomes has revealed nine other human-specific frameshift mutations (pseudogenization) leading to the mRNA decay or carboxy-terminal protein truncation [41]. Gene losses that occurred after human-chimpanzee divergence could play a role in adaptive evolution, as shown for caspase 12 inactivation during hominization [42]. In this framework, it is intriguing that a member of the hair keratin gene complex, *KRTHAP1*, and a serine protease inhibitor gene, *SERPINA13*, are also among the few genes specifically inactivated in human by a mutation [43,44].

In addition, we sequenced ORESTES for *CTAGE5 *and *CNOT6L *functional retrogenes, which are specific for primate and hominoid lineages, respectively. Moreover, the *CTAGE5 *retrogene is included in a larger, human-specific insertion (data not shown). It is indeed known that new genes have emerged after a burst of retroposition in primates [45]. Not surprisingly, this suggests that modifications of genes expressed in the skin participate in obvious differences between human and chimpanzee.

### Real-time PCR experiments

Real-time PCR experiments were used to measure the relative expression ratio of selected genes in the T4 (mainly GKs) and T1 (basal layer) fractions. In initial experiments using the *SCCE/KLK7 *gene as a GK specific marker, the T4/T1 ratios (54-189; Table 1) indicated that the T4 fraction was indeed highly enriched in GKs. However, subsequent experiments led to even higher ratios (for example, 800 for *FLG2 *and *GSDM*). Our approach thus led to the discovery of new, exquisitely specific gene markers for GKs that constitute valuable tools for detailed studies of epidermis architecture by histochemistry and *in situ *hybridization methods. Moreover, such highly specific expression strongly suggests that the corresponding genes play key roles in barrier function. The real-time PCR studies also revealed equal expression of several genes in the T4 and T1 fractions, suggesting that they are transcribed during all steps of keratinocyte differentiation. In addition to providing one with a highly purified GK fraction, our human skin fractionation method thus constitutes a new tool for the characterization of genes involved in the successive steps of terminal differentiation in the epidermis.

### GK-specific candidate genes

To further characterize genes poorly represented in databases, the 330 genes with the lowest EST number in the UniGene database (≤100 ESTs) were analyzed in more detail. Among these, the known specific genes involved in keratinocyte terminal differentiation account for only 15% of the panel, whereas 42% (139) encode hypothetical proteins. This shows that genes expressed specifically in the uppermost layers of the epidermis are poorly represented in the sequence databases, and suggests that some genes encoding hypothetical proteins may play a functional role in the late steps of epidermal differentiation. We specially focused on genes potentially involved in desquamation regulation as well as lipid metabolism and transport, considering their importance for barrier function establishment. Thus, 73 candidate genes were chosen for a quantitative study of their expression in the course of epidermal differentiation by real-time RT-PCR. The T4/T1 ratio for 20 of them could not be calculated by quantitative RT-PCR using the SYBR Green method, due to a very low expression level. This underlines the sensitivity of the ORESTES method in detecting rare transcripts. Among the 52 remaining genes, real-time RT-PCR experiments revealed that 33 are upregulated during late epidermis differentiation, and hypotheses on the function of some of them are presented below. Moreover, the expression pattern of 6 of these genes is mostly restricted to epidermis as shown by PCR on a panel of 16 human cDNAs prepared from various organs (Figure 4). Among the 330 genes poorly represented in EST databases that we identified, a significant proportion of granular keratinocyte specific genes are thus suspected to be present.

### Expression of proteases and protease inhibitors in the human epidermis

The balance between proteases and protease inhibitors is essential to desquamation [46]. The inactivation of the protease inhibitor cystatin M/E gene in mice causes lethality and defects in epidermal cornification [47]. Mutations in the *SPINK5 *gene (encoding another protease inhibitor) are responsible for Netherton syndrome (OMIM #256500), characterized by ichthyosiform erythroderma, bamboo hair and atopic dermatitis. In this framework, we identified three protease inhibitors potentially involved in desquamation.

In addition to the serine protease inhibitor *SERPINA12 *discussed above, we identified two other members of the serpin superfamily, *SERPINB7 *and *SERPINB12*, whose expression in the epidermis is reported here for the first time. Moreover, our real-time PCR experiments clearly show that these two protease inhibitors (as well as *SERPINA12*) are overexpressed in the uppermost epidermal layers. *SERPINB7*, also known as Megsin, is deposited in the extracellular matrix by kidney mesangial cells [48], but its targets have not been identified to date. *SERPINB12 *is expressed in many tissues, and displays inhibitor activity against trypsin-like serine proteases [49]. To understand the roles of these protease inhibitors in desquamation, it is of key interest to determine their molecular targets. Proteases expressed in the skin and potentially involved in desquamation are interesting candidates. Our ORESTES data set includes the serine protease kallikrein 7 (*SCCE*), which plays a key function in desquamation by cleaving two corneodesmosome components, desmocollin 1 and corneodesmosin [50]. Surprisingly, we did not detect kallikrein 5 (*SCTE*), which cleaves another corneodesmosome component, desmoglein 1. However, we detected for the first time the transcription of the *P11 *gene in the epidermis. This gene encodes a secreted serine protease previously shown to be expressed in the human placenta and various neoplasms of the breast, ovary, testis, and stomach [51]. Its specific targets remain unknown. In epidermis, *P11 *is upregulated in the granular layer with a T4/T1 ratio of 35 (Table 7). We suggest that P11 could act in the course of desquamation, either by cleaving corneodesmosome components, or by activating other proteases.

Protease inhibitors might also play a documented role in protecting the body from infection. In addition to the liver-expressed antimicrobial peptide *LEAP-2*, we detected the expression of the *WAP1 *and *WAP2 *genes, which encode serine protease inhibitors with antimicrobial activity in mouse tongue and kidney [52]. *WAP1 *appears to be expressed in all epidermal layers, whereas *WAP2 *is overexpressed in GKs and could thus play an antimicrobial role in uppermost epidermal layers. Therefore, our study contributes to enlarge the panel of proteases and protease inhibitors potentially involved in barrier function, regulation of desquamation and defense against microorganisms.

### Genes involved in lipid metabolism

Mutations of genes involved in various aspects of lipid metabolism are at the origin of several human genodermatoses (Table 8), underlying the key interest in the identification of new, lipid-processing genes expressed in the skin. We identified three new human genes, *LIPK*, *LIPM *and *LIPN*, which encode proteins containing two characteristic domains, the α/β hydrolase fold and the abhydrolase associated lipase region. They furthermore contain the consensus pattern of the active domain [53], suggesting that they are *bona fide *lipase genes (Figure 5b; Additional data file 4). The *LIPK*, *LIPM *and *LIPN *genes are strongly specific for the epidermis (Figure 4), and real-time RT-PCR experiments revealed a highly specific expression in GKs, with T4/T1 ratios >100.

**Table 8:** Monogenic diseases due to mutations of genes involved in lipid metabolism and displaying an epidermal phenotype

Gene	Function	MIM/reference	Pathology
*ABCA12*	ABC lipid transporter	#242500	Harlequin ichthyosis
*STS*	Steroid sulfatase	+38100	X-linked ichthyosis
*GBA*	Glucocerebrosidase	#230800	Gaucher disease
*ALOXE3*/*ALOXB12*	Arachidonate lipoxygenases	#242100	Non-bullous congenital ichthyosiform erythroderma
*CGI58 *(*ABHD5*)	Putative triglyceride lipase	[62]	Chanarin-Dorman syndrome
*LIPH*	Phospholipase A1	[63]	Hair growth defect

Phylogenetic studies showed that the LIPJ, LIPK, LIPM and LIPN proteins are very close to well-characterized members of the family LIPA and LIPF, both also encoded in the 10q23.31 locus. LIPA is a ubiquitous lysosomal cholesterol ester hydrolase (EC 3.1.1.13) [54,55], while LIPF is a secreted triglyceride lipase (EC 3.1.1.3) [56]. Interestingly, both LIPA and LIPF have a low pH optimum, in agreement with the acidic pH of the extracellular space in the stratum corneum [57,58].

The extracellular hydrolysis of triglycerides in free fatty acids and glycerol is essential to stratum corneum hydration [59]. Furthermore, long chain free fatty acids represent 16% of extracellular lipids, cholesterol esters 15%, and free cholesterol 32% [60]. Triglyceride lipase enzymatic activity has been detected in the intercellular space of the human stratum corneum [61], and little is known regarding the metabolism and extracellular modifications of cholesterol esters. The LIPK, LIPM and LIPN proteins are most probably secreted and could thus participate in the establishment of the barrier function by catalyzing the maturation of extracellular lipids. The weakly expressed, non-secreted LIPJ protein might play a distinct role.

The *LIPK*, *LIPM *and *LIPN*, but not *LIPJ*, gene orthologues could be identified in several vertebrate species, including mouse and rat. Surprisingly, a cluster of tandem duplicated genes encoding new lipases resides in the mouse and rat genomes, which could eventually increase the lipase repertoire of these species.

The new *LIPK*, *LIPM *and *LIPN *lipide hydrolase genes may play an essential function in lipid metabolism of the most differentiated epidermal layers, and are thus interesting gene candidates for genodermatoses of unknown origin. Accordingly, mutations in the *CGI58/ABHD5 *gene, which encodes a putative triacylglycerol lipase, are responsible for the Chanarin-Dorman syndrome, a neutral-lipid storage disease with ichthyosis [62]. Mutations in the *LIPH *gene are responsible for a hair growth defect [63]. Both CGI58/ABHD5 and LIPH proteins resemble the lipases encoded by genes from the 10q23.31 locus as they include an α/β hydrolase fold, but they lack the abhydrolase associated lipase region.

In addition to the lipases from the 10q23.31 locus, nine additional genes, predicted to be involved in lipid metabolism, were overexpressed in GKs (Table 8). Their homology with known genes suggests that they might act on fatty acid (*PNPLA1*, *THEM5*, *ADHD9*, *FAM83F*) or ceramide (*LASS3*, *ASAH3*, *SMPD3*) metabolism. Compared to other tissues, lipid metabolism in the epidermis presents many distinct characteristics, as it mainly occurs in the extracellular space. Extracellular lipids play key roles in the barrier function, particularly in hydrophobicity of the skin surface. Our study thus unraveled new actors in this particularly important process, and might shed new light on the etiology of genodermatoses.

### Genes of miscellaneous function

In addition to genes involved in protein degradation and lipid metabolism, we characterized new genes that might be of key importance in skin function. CASZ1 is a transcription factor induced during embryogenesis in the course of mesenchyme differentiation [64]. As its gene targets are presently unknown, its relatively high expression level in the human skin (20 ORESTES) calls for detailed functional studies in this tissue. Finally, eight genes encoding hypothetical proteins without known domains, such as *C20orf95*, *CXorf33*, or *LOC440449*, also displayed GK-specific expression. Their roles in epidermal differentiation remain completely elusive.

## Conclusion

We have described an original and efficient method for purifying GKs from healthy human epidermis. It is now of key interest to adapt this technique to biopsy fragments from patients suffering from various genodermatoses. The GK is the last cell type in the skin to display transcription activity before cornification, and we describe the expression of 3,387 genes, a proportion of which are expressed in GKs in a highly specific manner. We presume that many of them are important for the establishment of the barrier function, and as such deserve detailed functional studies. Moreover, we provide the scientific community with a list of gene candidates for genodermatoses of unknown origin. In particular, the understanding of complex diseases associated with defects in barrier function, such as psoriasis or atopic dermatitis, might benefit from identification of new epidermis-expressed genes located in associated loci. Among the genes described, some fit with, and even improve, our present knowledge of the barrier function, in particular concerning the fine-tuning of protein degradation and lipid metabolism. The precise function of the corresponding proteins will be assessed using mouse models and immunochemistry methods starting from healthy and pathological human skin. However, many of the genes described herein, often specifically expressed in GKs at high levels, encode putative proteins whose functions are totally obscure but that might well participate in the establishment of the skin barrier. Incidentally, we characterized a new gene, *C1orf81*, which is specifically inactivated or truncated in humans. Whether this gene loss participated in the establishment of the human species, and thus fits the 'less-is-more' hypothesis [65] remains a fascinating question. Our study of the human GK transcriptome thus opens new avenues for future research in many fields, including the normal functioning of the epidermis, the origin of genodermatoses, and even the emergence of the human species.

## Materials and methods

### Skin samples and RNA extraction

Normal human skin was obtained from patients undergoing abdominal plastic surgery (kindly provided by Professor JP Chavoin, "Service de Chirurgie Plastique et des Brûlés", Centre Hospitalier Universitaire Rangueil, Toulouse, France) after informed consent and in accordance with Helsinki principles. Subcutaneous fat was promptly removed and strips of skin were incubated, epidermis side up, for 1 h at 4°C in phosphate-buffered saline (PBS) containing 0.5 mg/ml thermolysin (T7902, Sigma, St Louis, MO, USA). The epidermis was dissected free of dermis tissue with forceps and rinsed in cold PBS. Epidermal fragments were either immediately frozen for total RNA extraction or incubated in 1× trypsin-EDTA solution (25300-054, Invitrogen, Carlsbad, CA, USA) at 4°C under gentle agitation for 15 minutes. The remaining epidermal fragments were rinsed in cold PBS and incubated in another trypsin-EDTA solution, while fetal calf serum (10270098, Invitrogen) was added to the suspended cells (10% final concentration). After centrifugation, the cells were frozen as dry pellets. The procedure was repeated twice, leading to three successive fractions of dissociated cells named T1, T2 and T3. The residual fragments (T4 fraction) were drained on a gauze compress, frozen and ground to a powder under liquid nitrogen. Total RNA was extracted from the various cell batches using the RNeasy extraction kit (Qiagen, Hilden, Germany). Purification of poly(A)+ RNA was always performed from the T4 fraction of individual patients, using oligo(dT)25-tagged magnetic beads according to the manufacturer's instructions (Dynal, Oslo, Norway). Two rounds of hybridization to the beads were performed. The mRNAs were treated with DNAse I (Invitrogen) and the absence of genomic DNA was confirmed by PCR using primers for the corneodesmosin gene (GenBank: AF03130).

### Morphological analysis of epidermis samples

After each trypsin incubation, an aliquot of epidermal fragments was fixed in Bouin's solution, embedded in paraffin, and sections (10 μm) were stained with hematoxylin-eosin.

### Production and analysis of ORESTES

ORESTES production was essentially performed as described [66]. Purified mRNA (20 ng) was heated 10 minutes at 65°C, reverse transcribed at 37°C for 1 h with 200 U of Moloney murine leukemia virus reverse transcriptase (Promega, Madison, WI, USA) and 10 pmol of an arbitrary selected primer (18-25 nt) in a final volume of 20 μl. The reaction products (1 μl) were amplified by PCR using either the primer used for the reverse transcription, or a single, alternative arbitrary chosen primer. The hybridization step of the first PCR cycle was set at 37°C, while the 35 remaining cycles were performed in standard conditions with a hybridization temperature complying with the length of the primer (typically 55°C). After gel electrophoresis, products with predominant bands reflecting the amplification of highly abundant sequences were not further processed. Smear-like reaction products were gel-purified with a 500 bp cut-off. Mini-libraries were then produced by T/A cloning of the purified PCR fragments (TOPO-TA cloning kit, Invitrogen).

### Plasmid purification and sequencing

Sequencing was performed by standard procedures (ABI Prism Dye Terminator Cycle Sequencing kit, Applera, Norwalk, CI, USA) after either plasmid purification (Wizard miniprep kit, Promega) or rolling circle amplification of the plasmids.

### Sequence analysis

An automated protocol for the sequence analysis was used to verify sequence quality. Sequences were then analyzed using a stepwise approach. Starting with RepeatMasker [67], ORESTES were clustered with the PHRAP algorithm [68]. Consensus sequences or singletons were annotated using BLAST searches against human databases (best-hit among successively RefSeq, Uniprot and EST_Human databases [69]). Sequences were also aligned on the human genome (May 2004 assembly) using BLAT [18] and inserted as a custom track into the UCSC Genome Browser.

### Analysis of gene expression

For quantitative real-time RT-PCR experiments, all primer pairs (available upon request) were chosen to generate amplicons of 100-250 bp encompassing different exons, thus avoiding the amplification of potential contaminating genomic DNA. The primer sequences were designed using Primer3 software [70] and BLAST analysis [69] ensured the absence of similarity to any other human sequence. Reverse transcription was performed by standard procedures, starting from 100 ng of total RNA of each cell batch and using a mixture of oligo(dT) and random hexamers. Amplification assays were performed with the ABI prism 7000 Sequence Detection System and analyzed with the corresponding software (Applied Biosystems, Foster City, CA, USA) using the qPCR ROX-&GO Green mix (MP Biomedicals, Irvine, CA, USA). Fluorescence was quantified as Ct (threshold cycle) values. Samples were analyzed in triplicate, with differences between the three Ct values lower than 0.3. Expression levels were calibrated using *galectin 7 *(*LGALS7*), or *beta-2-microglubulin *(*B2M*) mRNA as internal controls. The differences between the mean Ct values of the various amplicons and the reference genes are denoted (ΔCt). The difference between ΔCt obtained with the indicated cell samples are labeled ΔΔCt; 2^ΔΔCt ^gave the relative level of gene expression between the T1 and T4 fractions. Control wells containing the SYBR Green PCR master mix and primers without template cDNA emitted no significant fluorescence after 40 cycles.

Human Multiple Tissue cDNA panels I and II obtained from Clontech (Palo Alto, CA, USA) were used as templates for PCR analysis. A control reaction with T4 cDNAs was carried out in parallel. The reactions were conducted for 35 cycles in standard conditions. The PCR products were separated on 1.5% agarose-TAE gels.

### RACE-PCR experiments

We performed 5' RNA ligase-mediated (RLM)-RACE using the FirstChoice RLM-RACE kit (Ambion, Austin, TX, USA). Briefly, total RNA was dephosphorylated with calf intestine phosphatase then decapped using tobacco acid pyrophosphatase to target full-length mRNA. An adapter was then ligated to mRNA and reverse transcription was performed using random decamers. PCR was performed to amplify the resulting cDNA using the Outer 5' RLM-RACE primer and a gene specific lower primer. Nested PCR was then performed with the Inner 5' RLM-RACE primer. The RACE nested PCR products were cloned into the pCRII-TOPO vector using a TOPO T/A cloning kit (Invitrogen) and sequenced.

### NCBI gene ID references

*GAPDH*, 2597; *SOD1*, 6647; *ACTB*, 60; *B2M*, 567; *HPRT1*, 3251; *HMBS*, 3145; *TBP*, 6908; *UBC*, 7316; *PNAS-123*, 85028; *PPP2R5A*, 5525; *RPS11*, 6205; *RPS12*, 6206; *RPL10*, 6134; *EIF4A1*, 1973; *KRTHAP1*, 8686.

## Additional data files

The following additional data are available with the online version of this paper. Additional data file 1 is a screen copy of the ORESTES custom track of the human UCSC Genome Browser that shows the genomic localization of the newly described *C1orf81 *gene with the corresponding ORESTES (chr1:199,164,695-199,202,419; hg18, March 2006). Additional data file 2 is a table of genes (with 100 or less UniGene ESTs) expressed in various tissues but for which epidermal expression had never been assessed. Additional data file 3 is a colored text file containing the alignment of human and rhesus genomic sequences showing the location of the insertion point and tandem site duplication for the hominoid-specific CNOT6L processed retrogene. Additional data file 4 is the alignment of protein sequences of the ten mouse lipases, including Lipdc1-5. The alignment was generated with Multalin software [71]; the amino acids of the catalytic triad are boxed. Additional data file 5 is a table providing gene nomenclature and IDs for five new mouse lipase genes.

## Supplementary Material

Additional data file 1 Screen copy of the ORESTES custom track of the human UCSC Genome Browser that shows the genomic localization of the newly described *C1orf81 *gene with the corresponding ORESTES (chr1:199,164,695-199,202,419; hg18, March 2006). Click here for file

Additional data file 2 Genes with 100 or less UniGene ESTs expressed in various tissues but for which epidermal expression had never been assessed. Click here for file

Additional data file 3 Alignment of human and rhesus genomic sequences showing the location of the insertion point and tandem site duplication for the hominoid-specific CNOT6L processed retrogene. Click here for file

Additional data file 4 The alignment was generated with Multalin software [71]; the amino acids of the catalytic triad are boxed. Click here for file

Additional data file 5 Gene nomenclature and IDs for five new mouse lipase genes. Click here for file
