# Supplementary material for: Large-scale identification of human genes implicated in epidermal barrier function
Source: Genome Biol. 2007 Jun 11;8(6):R107. doi: 10.1186/gb-2007-8-6-r107 (PMC2394760; doi:10.1186/gb-2007-8-6-r107)
Supplement: Additional data file 2 — Genes with 100 or less UniGene ESTs expressed in various tissues but for which epidermal expression had never been assessed. [file gb-2007-8-6-r107-S2.rtf]

Additional data file 2: Genes with 100 or less UniGene EST expressed in various tissues for which epidermal expression had never been assessed.

ORESTES	Gene Symbol	UniGene EST	Full name	
1	WFDC12	3	WAP four-disulfide core domain 12	
6	SERPINB12	6	serpin peptidase inhibitor, clade B, member 12	
4	ROH1	9	ROH1	
1	LOC401904	11	similar to 60S ribosomal protein L23a	
2	WNT16	11	wingless-type MMTV integration site family, member 16	
1	LOC388574	12	similar to 60S ribosomal protein L23a	
4	BPIL2	13	bactericidal/permeability-increasing protein-like 2	
2	RAET1E	16	retinoic acid early transcript 1E	
2	CD207	17	CD207 antigen, langerin	
13	LY6G6C	19	lymphocyte antigen 6 complex, locus G6C	
1	SOAT2	21	sterol O-acyltransferase 2	
2	FAM10A5	24	family with sequence similarity 10, member A5	
1	ZNF396	26	zinc finger protein 396	
1	RPS6KA6	28	ribosomal protein S6 kinase, polypeptide 6	
1	PASD1	29	PAS domain containing 1	
34	LOC400590	30	hypothetical LOC400590	
11	SLC15A1	30	solute carrier family 15, member 1	
1	XKRX	31	XK, Kell blood group complex subunit-related, X-linked	
1	ANKRD20A	31	ankyrin repeat domain 20 family, member A1	
6	OR2A20P	34	olfactory receptor, family 2, subfamily A, member 20 pseudogene	
1	HAVCR1	34	hepatitis A virus cellular receptor 1	
5	RHOV	36	ras homolog gene family, member V	
1	INCA1	38	inhibitor of CDK interacting with cyclin A1	
1	BNC1	39	basonuclin 1	
1	CARD15	39	caspase recruitment domain family, member 15	
3	LGALS7	40	lectin, galactoside-binding, soluble, 7 (galectin 7)	
1	IL22RA1	40	interleukin 22 receptor, alpha 1	
4	SERPINB13	42	serpin peptidase inhibitor, clade B, member 13	
1	NIP	45	homolog of Drosophila Numb-interacting protein	
2	ATG9B	46	ATG9 autophagy related 9 homolog B 	
5	MAPK15	47	mitogen-activated protein kinase 15	
5	CCL22	48	chemokine (C-C motif) ligand 22	
1	ACCN3	48	amiloride-sensitive cation channel 3	
2	RNF39	48	ring finger protein 39	
5	GJB5	50	gap junction protein, beta 5 (connexin 31.1)	
1	GGTA1	50	glycoprotein, alpha-galactosyltransferase 1	
2	SOX15	51	SRY (sex determining region Y)-box 15	
2	SFTPD	53	surfactant, pulmonary-associated protein D	
1	RP11-19J3.3	54	hypothetical protein MGC88047	
4	GSTT2	55	glutathione S-transferase theta 2	
2	GRHL3	56	grainyhead-like 3 	
2	BNIPL	58	BCL2/adenovirus E1B interacting protein like	
1	KLC2L	60	kinesin light chain 3	
2	BLOC1S3	61	biogenesis of lysosome-related organelles complex-1, subunit 3	
1	PDE4C	63	phosphodiesterase 4C, cAMP-specific 	
2	SLC6A9	63	solute carrier family 6, member 9	
1	GIPC2	64	GIPC PDZ domain containing family, member 2	
2	CBLC	65	Cas-Br-M ecotropic retroviral transforming sequence c	
2	IPMK	65	inositol polyphosphate multikinase	
5	USP43	67	ubiquitin specific peptidase 43	
1	HYPE	67	Huntingtin interacting protein E	
2	SLC37A2	67	solute carrier family 37, member 2	
1	HCN1	69	hyperpolarization activated cyclic nucleotide-gated potassium channel 1	
1	TXK	71	TXK tyrosine kinase	
3	MYCL1	72	v-myc myelocytomatosis viral oncogene homolog 1, lung carcinoma derived 	
1	LGR6	72	leucine-rich repeat-containing G protein-coupled receptor 6	
1	LOC440582	73	similar to Peptidyl-prolyl cis-trans isomerase E (Cyclophilin 33)	
1	HLA-DQB2	74	major histocompatibility complex, class II, DQ beta 2	
2	FBXO27	74	F-box protein 27	
6	CCL27	75	chemokine (C-C motif) ligand 27	
1	MSP	75	transmembrane protease, serine 13	
5	GCET2	75	germinal center expressed transcript 2	
1	NEIL3	76	nei endonuclease VIII-like 3 	
1	SLC6A16	77	solute carrier family 6, member 16	
2	TMEM16H	78	transmembrane protein 16H	
4	USF1	79	upstream transcription factor 1	
3	RAB38	79	RAB38, member RAS oncogene family	
1	CBR3	79	carbonyl reductase 3	
1	TLR3	81	toll-like receptor 3	
4	CHMP4C	81	chromatin modifying protein 4C	
1	FBF-1	81	Fas binding factor 1	
1	RNASEL	81	ribonuclease L 	
1	ENTPD7	83	ectonucleoside triphosphate diphosphohydrolase 7	
2	GPR39	85	G protein-coupled receptor 39	
1	CARD14	86	caspase recruitment domain family, member 14	
4	COL5A3	86	collagen, type V, alpha 3	
1	TRAF3IP1	86	TNF receptor-associated factor 3 interacting protein 1	
1	TRMT5	86	TRM5 tRNA methyltransferase 5 homolog 	
1	AFMID	87	arylformamidase	
2	GAL3ST4	87	galactose-3-O-sulfotransferase 4	
1	PROM2	88	prominin 2	
1	PTK6	88	PTK6 protein tyrosine kinase 6	
2	TNFRSF10A	88	tumor necrosis factor receptor superfamily, member 10a	
1	IL7	89	interleukin 7	
3	PLXDC2	89	plexin domain containing 2	
1	IGSF9	90	immunoglobulin superfamily, member 9	
3	PUS3	90	pseudouridylate synthase 3	
1	DSCR1L2	91	Down syndrome critical region gene 1-like 2	
2	N4BP3	91	Nedd4 binding protein 3	
2	RGR	91	Ral-GDS related protein Rgr	
1	AP4E1	91	adaptor-related protein complex 4, epsilon 1 subunit	
1	CHRNB1	92	cholinergic receptor, nicotinic, beta 1 	
1	FRAT2	92	frequently rearranged in advanced T-cell lymphomas 2	
2	ADAMTS17	93	ADAM metallopeptidase with thrombospondin type 1 motif, 17	
1	SLC6A17	93	solute carrier family 6, member 17	
1	SURF2	93	surfeit 2	
3	ANXA9	93	annexin A9	
1	DNAH9	94	dynein, axonemal, heavy polypeptide 9	
1	ESPN	94	espin	
2	JPH2	94	junctophilin 2	
1	ARHGEF5	94	Rho guanine nucleotide exchange factor 5	
2	LOC283874	95	hypothetical protein LOC283874	
2	SLC9A3	96	solute carrier family 9, member 3	
1	LTB4R	96	leukotriene B4 receptor	
1	SLC30A4	98	solute carrier family 30, member 4	
1	HRH1	98	histamine receptor H1	
2	PIK3C2G	98	phosphoinositide-3-kinase, class 2, gamma polypeptide	
3	TRAF6	98	TNF receptor-associated factor 6	
1	CD96	99	CD96 antigen	
1	RASGEF1B	99	RasGEF domain family, member 1B	
3	DUOX1	100	dual oxidase 1	
