# Supplementary material for: Large-scale identification of human genes implicated in epidermal barrier function
Source: Genome Biol. 2007 Jun 11;8(6):R107. doi: 10.1186/gb-2007-8-6-r107 (PMC2394760; doi:10.1186/gb-2007-8-6-r107)
Supplement: Additional data file 3 — Alignment of human and rhesus genomic sequences showing the location of the insertion point and tandem site duplication for the hominoid-specific CNOT6L processed retrogene. [file gb-2007-8-6-r107-S3.rtf]

Additional data file 3: Hominoid-specific CNOT6L processed retrogene.
The alignment of human and rhesus genomic sequences was retrieved from the Rhesus Alignment Net track of the UCSC Genome Browser, and allowed to precisely localize the insertion point and Tandem Site Duplication (TSD), as described (Weber, 2006).
This hominoid-specific insert (TSD boxed) contains a processed retrocopy of the CNOT6L gene (blue letters, 11 exons, stars indicate exon-exon junctions), followed by a human-specific AluYa5 insertion (underlined, TSD highlighted in green). The TSD corresponding to the CNOT6L retrogene and the Alu element are largely overlapping. The sequence corresponding to ORESTE AX43 is highlighted in yellow. The coordinates of sequences shown are:
human: chr15:54,082,391-54,086,959 (March 2006)
rhesus: chr7:34,329,457-34,329,575 (Jan. 2006)

rhesus ATCCTTAATATCCTTAGTGCAACCAAGAACAGTAGAATGTTTAAAGAAGAATGTT 
human  ATCCTTAATATCCTTAGTGCAACCAAAAACGCTAGAATGTTTAAAGAAGAATGTT 
       ************************** ***  ***********************

gatgtctttgctgccctcagccccgcctcccccgccgccgccgcctcccccgccgccgccgcctcccccgccgccgccgcctcccccgccgccgccgcctcccccgccgccgccgcctcccccgccgccgcagcaccagcaccagccgcggcagcaccagcaccagccgccgcagcaccagcaccagccgccccatcgccaccgccgccgccgccgcccggatcctggcgcgctgaatgcag*actaacagggatgccaaaggaaaaatatgatcctccagatcctcgcagaatttataccatcatgtcagcagaggaggtagccaatgggaaaaaatctcactgggcagaattagaaatctcgg*ggaattgcttgttacgggttttgccttatgaacttggtgggctcttccagctacaaactctaggtttgaaag*gcaatcctttatcacaggatattctcaacttataccaggacccagatggaacccgaaatctactgaacttcatgcttgacaatctcgcag*ttcatccagagcagcttcctccgaggccatggattacattaaaagaacgagaccaaattctgccatcag*catcattcacggttatctgttacaatgtgttatgtgataaatacgctacccggcagctatatggttattgcccatcctgggcattaaacttggaatacaggaaaaagggaattatggaagaaattgttaactgtgacgcagatatcattagtcttcag*gaagtggaaacagagcaatacttcactctctttctgccagcattgaaggagcgtgaatatgatggatttttttctccaaagtcacgtgccaaaatcatgtctgagcaggagagaaagcatgtagatggttgtgcaatatttttcaaaagagaaaa*atttacattggtgcagaagcatgcagtggaatttaaccaagtggcaatggctaattcagatggatccgaagctatgctgaacagagtgatgacaaaagataacattggtgtcactgtggtattagaggtccacaaagaactatttggagcag*gtatgaagcctattcatgctgcagacaaacagctgcttatagtggcaaatgcccacatgcattgggacccagagtattctgatgtgaagctcatccagaccatgatgtttgtctcagaggttaaaaccattctggagaaagcctctagtaggtctggcagcccaactgcagatcctaattccatcccgctggtgctatgtgcagatcttaactcattgccagattcag*gtgttgtggaatacttaagcaatggaggaatagctgacaaccataaagacttcaaggaactaaggtacaataagtgtcttatgaacttcagctgcaatggaaagaatggaagctcagaagggagaatcacacatggcttccaacttaagagcgcctacgaaaataacttgatgccttacaccaattacacctttgatttcaaa*ggcgtgattgactacattttctattccaagactcatatgaatgtgcttggtgtcctggggcctttagatcctcaatggctggttgagaacaacatcactgggtgtccacaccctcacatcccttcagaccacttctcactgttaacacaacttgaactccaccctccactcctgcctcttgtcaatggtgttcacttgcctaatcggaggtggtggagtactgccccgccaagacggggatctgttgctatggacctgtacagttgtaaatcaaagtatgtaggagtgaagtatggccatccttaagctgcttcttcacgtttctttctttatgtgtttgctgtaagactttgtacatttttgtgcatattggtatcatttggcagtagggctggaaccaaagtattactctctttccaaaattttaatttaacatgtttttaaattggaccttctttatattgtattaacagccagcattcaaaattgataaattaccaatttgaggcccaacaacagtgtatttgtttttccaaaacaaatactttcttttgaatggtttcagtgagccaaccattttataaaaggcaatttttaaaaactataaatacagtattttaaactgaatgatgatatgccttccagggaaaaccttgaatttttcctttataactgacttttggattcccaagtcatttgcacattaacagagtacttaaatttacttgttcagtacataaactatgatatagcctctatacatggaaagaaaatttgaaaagttaaagatggttgcacagtatgctttcataatcaagcatgtaacaccacatgacaatttgttggccaaatgctggtttggagtttttttgaggaatcactttggtttttttgtcctctctatataaccttattggaagtattaattctaagcctgtctctcaagtttatttatagagaaagagtaagtaatctgtattgccacataccttgaaaatagaatgcggtacgtttaggatgcccacagaatgaatttttccttattccaattcaaccttctgtctggttgtgccaggaaaacagatgttatatgacctatatcatttttgccattatagtcaaatgttaaaagaagaaaaaaatctgctgaataaaaaggccttgatcagagtttcagatggggaaaatcatacagatattttatgtgtttccaacacagattatgtggctgttggttttttgagatgacagtgcaaaggatttgggagagagaacaaatttggggcagtagttgaaaacgttgggtcattttcctgactctagctgccaaatggccatcatatgcttttaatctttgtttcagttgtctgtcagggtttaattaagaagctactggtttattcccaattgttgatgcctttaggtatgttggaatcttttttttgcctaggaggagccagttgaaaatctgtgactcaagaggcagtgaacagaatactgttttctggggaaaaatttgttggctacttgatgttaattatggcacagtaacaggaaaaggttgtgtctgtgtttttaagtttttctttattctgcttttttgctgctataagagttttctgaaatttatattttaaacttttcatgcactttactgtttctagtctcaaaatgtgatatttttaataaacaagaaattttccattatgtgaatgaaattttaaaagacaatagcctatatttgtgtctcactaatatataaagtacaggttaaatttaaattatttaattagttttaaatatacacaatttgtctcctctttcaaacctgacatcttaaactgttttattagtcttaaatgatgcatttactttagtcattttatgctaatttcttccatagtaaattaatcaggctatataaggtaatatttccccagagggtaattttagttggaggagggtggtgggatgatgtcatatcatacatgggattgcatcagaagggttctgtaaagcctgaactcccttttaaaagtgcctagtgatagaggcgttgtttgtcatctattataattggaatgtcattgtagttcagtgaattttgatgtaaataaaatatcttttaaaaatgttaaagtaccagaataaacaaacaaaaagaaacaacccttaagatgacagattttcctcaacatgcaggtttcccttcttatatacctcaagtatccaacatctagccatgcaaattgattacctgaagcatagtactagaaagtagaatagcatgaaaaacttaattttgtggacattacctttttttgtaatcagctactgtatgttttattttagatcttttgttttgggtgggttttctgctctggaggtatatgcattaacaaaacattttcctccttcatatatgcatgttaattagcaatgtaagcaatatttcctaccatacttcactcccaatatttttggagatgtttgtataaaatggaatttaaagttcacttatgattgtatatgaaccacagagaagcccatttattaataacaaacttttttaatagttaaatgtggaaggaaaaaataaaaatgggaaacattgtaaaaaaataaaaaaattaaaaaaaatgaagaggccgggcgcggtggctcacgcctgtaatcccagcactttgggaggccgaggcgggcggatcacgaggtcaggagatcgagaccatcccggctaaaacggtgaaaccccgtctctactaaaaatacaaaaaattagccgggcgtagtggcgggcgcctgtagtcccagctacttgggaggctgaggcaggagaatggcgtgaacccgggaggcggagcttgcagtgagccgagatcccgccactgcactccagcctgggcgacagagcaagactccgtctcaaaaaaaaaaaaaaaaaaatgaagaatgtt

human  TAAAATTGCTAACTTATAAGGACAGTTCTCTTCTAAAGAGGGTGCATTCTGTTATAAGAAAGTCC
rhesus TAAAATTGCTAACTTGCAAGGAGAGTTCTCTTCTAAAGACAGTGCATTCTGTT TAAGAAAGTCC 
       ***************  ***** ****************  ************ ***********
