# Supplementary material for: Large-scale identification of human genes implicated in epidermal barrier function
Source: Genome Biol. 2007 Jun 11;8(6):R107. doi: 10.1186/gb-2007-8-6-r107 (PMC2394760; doi:10.1186/gb-2007-8-6-r107)
Supplement: Additional data file 5 — Gene nomenclature and IDs for five new mouse lipase genes. [file gb-2007-8-6-r107-S5.rtf]

Additional data file 5: Gene nomenclature and IDs for five new mouse lipase genes.

Former entry	Gene Symbol	Gene Name	GeneID	MGI	
EG329055	Lipdc1	lipase domain containing 1	329055	3647308	
EG668114	Lipdc2	lipase domain containing 2	668114	3644466	
EG668106	Lipdc3	lipase domain containing 3	668106	3647649	
AI747699	Lipdc4	lipase domain containing 4	381236	2147592	
LOC628236	Lipdc5	lipase domain containing 5	628236	NONE yet	

 
